# The impact of environmental factors on marine turtle stranding rates

**DOI:** 10.1371/journal.pone.0182548

**Published:** 2017-08-03

**Authors:** Jaylene Flint, Mark Flint, Colin J. Limpus, Paul C. Mills

**Affiliations:** 1 *Veterinary*-Marine Animal Research, Teaching and Investigation (*Vet-*MARTI) Unit, School of Veterinary Science, The University of Queensland, Gatton, Queensland, Australia; 2 Department of Preventative Veterinary Medicine, College of Veterinary Medicine, The Ohio State University, Columbus, Ohio, United States of America; 3 Queensland Department of Environment and Heritage Protection, Queensland Government, Brisbane, Queensland, Australia; University of Minnesota, UNITED STATES

## Abstract

Globally, tropical and subtropical regions have experienced an increased frequency and intensity in extreme weather events, ranging from severe drought to protracted rain depressions and cyclones, these coincided with an increased number of marine turtles subsequently reported stranded. This study investigated the relationship between environmental variables and marine turtle stranding. The environmental variables examined in this study, in descending order of importance, were freshwater discharge, monthly mean maximum and minimum air temperatures, monthly average daily diurnal air temperature difference and rainfall for the latitudinal hotspots (-27°, -25°, -23°, -19°) along the Queensland coast as well as for major embayments within these blocks. This study found that marine turtle strandings can be linked to these environmental variables at different lag times (3–12 months), and that cumulative (months added together for maximum lag) and non-cumulative (single month only) effects cause different responses. Different latitudes also showed different responses of marine turtle strandings, both in response direction and timing.Cumulative effects of freshwater discharge in all latitudes resulted in increased strandings 10–12 months later. For latitudes -27°, -25° and -23° non-cumulative effects for discharge resulted in increased strandings 7–12 months later. Latitude -19° had different results for the non-cumulative bay with strandings reported earlier (3–6 months). Monthly mean maximum and minimum air temperatures, monthly average daily diurnal air temperature difference and rainfall had varying results for each examined latitude. This study will allow first responders and resource managers to be better equipped to deal with increased marine turtle stranding rates following extreme weather events.

## Introduction

In recent years, tropical and subtropical regions, such as Queensland, have experienced many extreme weather events [1–7], including droughts, cyclones and protracted rain depressions. In Australia, during summer there is a heightened risk of extreme weather and warmer temperatures, the summer of 2010/2011 in Queensland is of particular note. During this time, cyclones and protracted rain depressions caused wide-spread flooding which in turn led to increased periods of turbid water and increased nutrient and sediment loads from freshwater run-off being dumped into all four major coastal waterways (Brisbane, Fitzroy, Burnett and Burdekin Rivers) [8]. The cyclones and floods stressed coral reefs and seagrass beds causing large-scale die-off of ecologically important seagrass species and decreased water quality intermittently along the entire length of the Queensland coastline south from Cairns [8–11]. It was postulated that within a year (short-term) of these types of catastrophes, marine megafauna show an increase in the number of stranding, mortalities and exacerbated poor health conditions [12]. In a similar ilk, it has been shown that environmental variables affect seabird wrecks numbers and locations [13].

The ongoing poor weather conditions recently experienced are unprecedented in the 35 year history of the Great Barrier Reef Marine Park [11] and Queensland in general [14] since European settlement. The magnitude and scale of the bad weather conditions experienced during early 2011 on the Great Barrier Reef have not been seen since recording began in 1918 [11].

Norman et al. [15] stated that the ability to understand and investigate marine mammal unusual mortality events and other unexpected strandings that involve substantial die-offs of the marine mammal population are important events which serve as indicators of ocean health. This may give better insight into larger environmental issues, which can have implications for human health and animal welfare. This One Health paradigm can also be applied to marine turtle strandings as marine turtles have been proposed as sentinels of environmental health [16–18] and, as such, an increase in the numbers of animals which strand can indicate that the environments in which they live have changed [18].

It has been suggested that marine turtle stranding numbers follow seasonal trends influenced by weather events as well as land-based and at-sea seasonal activities. There have been links made between extreme weather and increased strandings within 12 month periods as outlined by [12,19–21].

Meager and Limpus [21] stated that the most plausible explanation for the high rate of strandings and mortalities of near shore green turtles during 2011 were extreme weather events that occurred in late 2010 and early 2011, which impacted on seagrass foraging areas. They linked this because most of the examined mortalities were attributed to protracted ill health/poor body condition in green turtles and dugongs; which both primarily forage on seagrass. There was evidence that seagrass pastures, mangrove forests, algal beds and coral reefs in Queensland were impacted by a combination of elevated rainfall, flooding and three cyclones (Category 5 Yasi, Category 2 Anthony and Category 1 Tasha) with a protracted low pressure system during the summer of 2010/2011.

This study examined marine turtle stranding rates in relation to environmental variables (including rainfall, freshwater discharge rates and air temperature). Different latitudinal blocks, species and age classes were investigated to determine if there were different responses. We summarized and analyzed the available data to provide first responders and management agencies with information to better assist them when responding to stranding events. The databases used for this study are the most comprehensive databases available for Queensland marine turtle records and was established over 30 years ago.

## Methods

### Stranding data

StrandNet is the Queensland Government’s Department of Environment and Heritage Protection (EHP) statewide database which records dead, sick and injured threatened marine animals for the entire coast of Queensland and adjacent Commonwealth waters. Records are received from members of the public, and employees of EHP, Queensland Parks and Wildlife (QPWS), Queensland Department of Agriculture, and Fisheries (DAF) and the Great Barrier Reef Marine Park Authority (GBRMPA). Information is collated and stored in this central database. Once reports are entered by on-ground staff the information available is verified by regional and state coordinators for standardization. Additional data is often obtained after the stranding event from veterinarians, pathologists and other biologists who complete more detailed post-mortem investigations.

As a proxy of age class, standard measurements such as curved carapace length (CCL) and tail to carapace length (TCL) were collected at the time of initial stranding [22]. This data was used to assign turtles into 3 age classes: small immatures, large immature and adult sized.

Sex was determined by gonad examination by trained personnel either onsite or using photographs or measurements [23,24].

Based on dichotomous key characteristics [25,26], species was determined as one of six turtle species including subspecies green (*Chelonia mydas)*, loggerhead (*Caretta caretta)*, flatback (*Natator depressus*), hawksbill (*Eretmochelys imbricata)*, leatherback *(Dermochelys coriacea)*, olive ridley *(Lepidochelys olivacea)*, black turtle *(Chelonia mydas agassizi*), as a hybrid animal or species unknown. Due to debate over species versus subspecies and a small dataset, we removed the black turtle from the individual species analyses.

**Fig 1:**
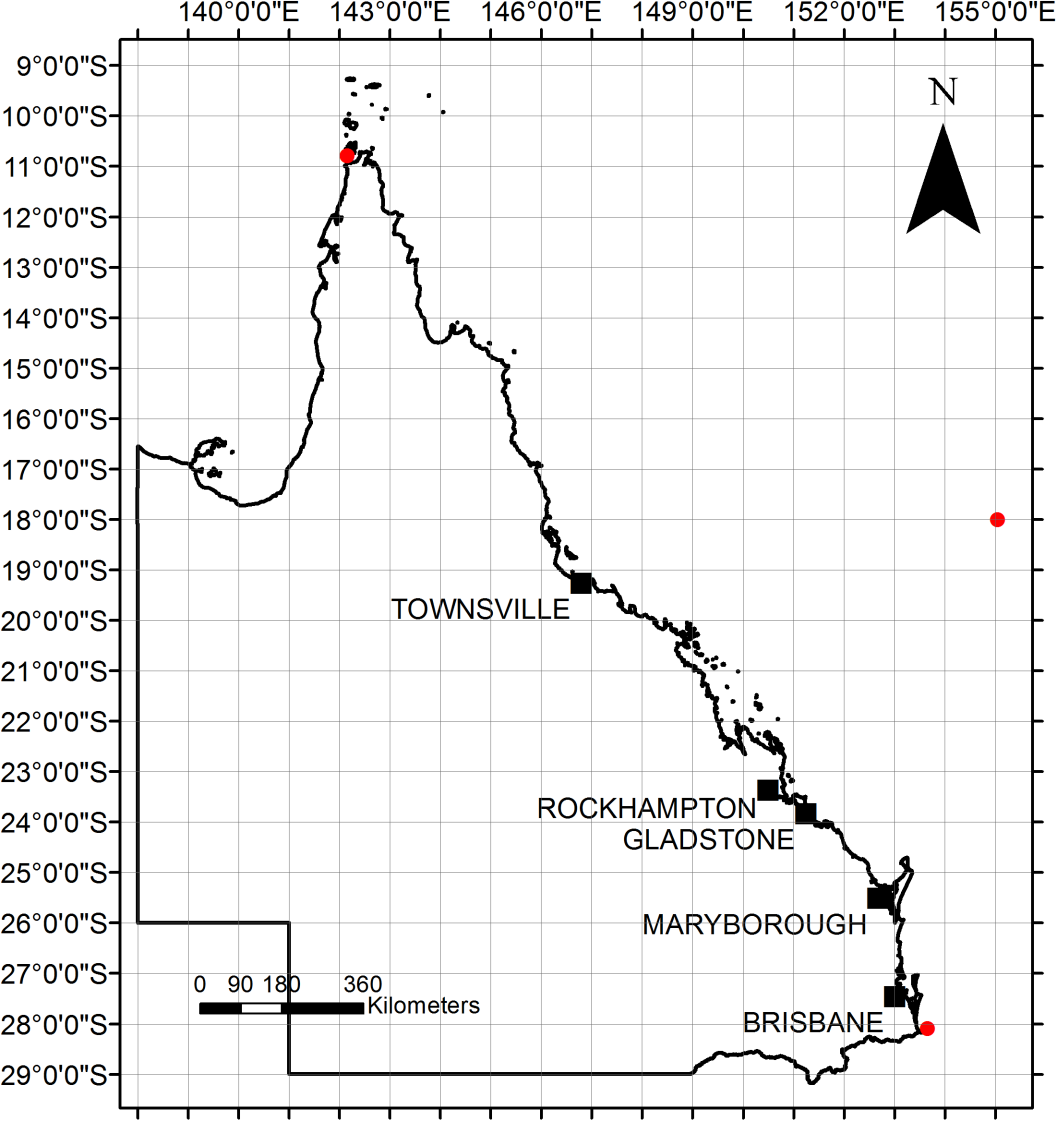
Map of Queensland coast. Red dots denote limits of study area.

**Fig 2:**
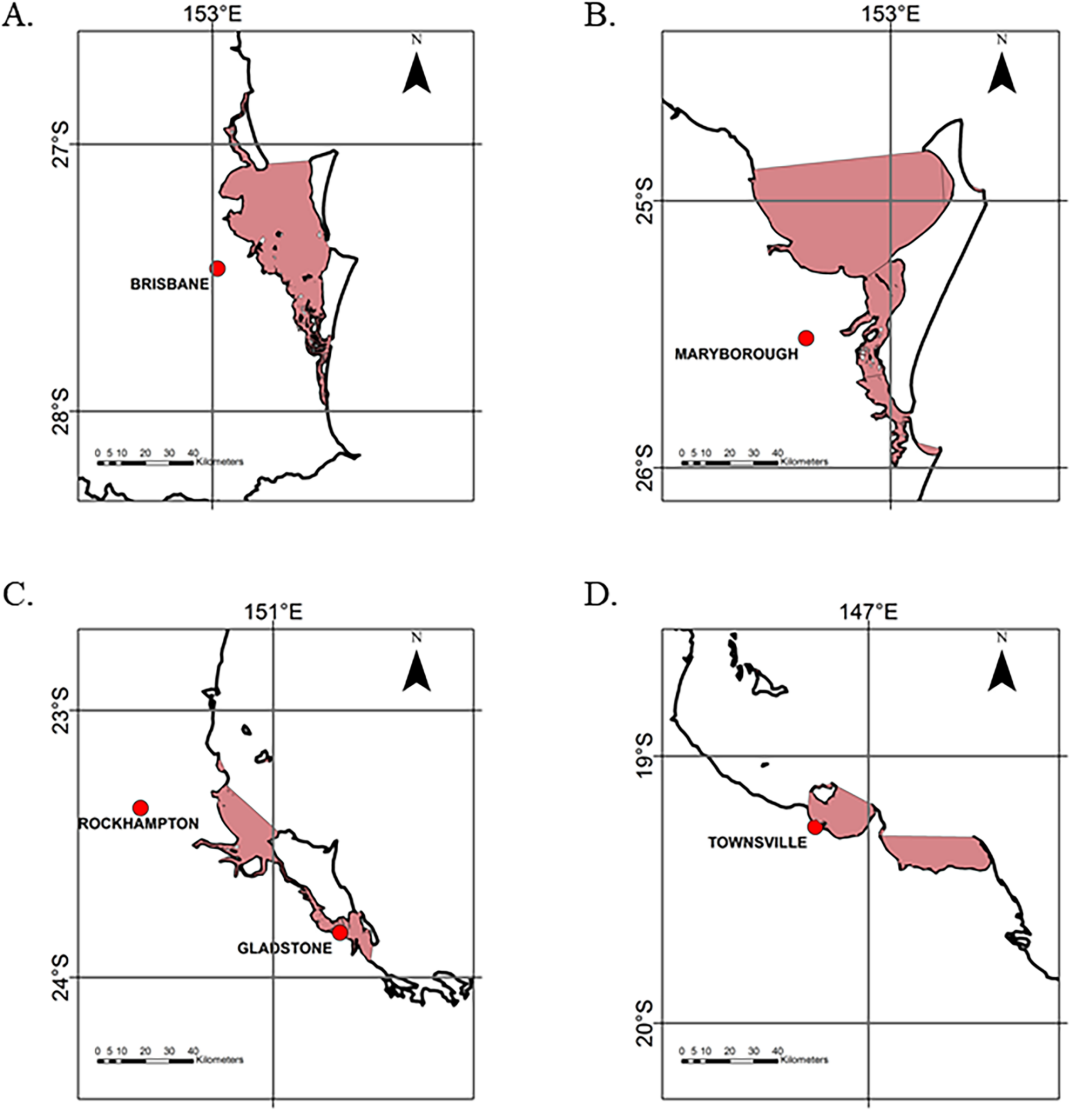
Embayments used for data analysis. 27°, B. 25°, C. 23°, D. 19°. Shaded area represents embayment areas used for analysis.

The study area encompassed latitude -10.78° to -28.16° and longitude 142.15° to 155° (**Fig 1**). This part of the east coast of Queensland was selected as it has a long-term and comprehensive dataset; with data collection biased to regions of survey and higher populations. This limitation is openly acknowledged by Meager and Limpus (2012) but considered valid as a representative of a minimum recovery rate and indicative of trends occurring. As the exact location where a stranding was reported was not necessarily where the impact/incident occurred, strandings were grouped into latitudinal blocks of 1° to account for this potential error. The areas of focus for this study were the hotspots recognized by Flint et al. [27] as -27°, -25°, -23° and -19° (**Figs 1 and 2**). In addition, major embayments, irrespective of latitudinal blocks were assessed (**Fig 2**).

#### Time

The date a turtle was reported stranded was used as a proxy of time of death, grouped to a monthly scale.

#### Cause of stranding

The term ‘stranding’ is used here to incorporate all reported sick, injured, incapacitated or dead marine turtles that were either found ashore or, in rare cases, were encountered at sea. It included turtles which were entangled in fishing nets, synthetic debris or rescued from a situation where they would have died had it not been for human intervention [28].

Within StrandNet, the primary cause of death/stranding was identified based on gross examination, photograph and/or necropsy by trained personnel [12,29]. The single cause of stranding identified in StrandNet was based on the summation of information available.

### Environmental data

Rainfall, freshwater discharge and air temperature were examined as environmental variables. These were selected as they provided the most comprehensive, readily available and up to date dataset of environmental conditions available. Turbidity, water temperature, pH and salinity were not used due to paucity of current available data along the Queensland coastline.

Freshwater discharge is the amount of freshwater running through a river’s gauging (recording) station, measured in cumecs (cubic meter per second, m^3^.s^-1^). Freshwater discharge data was downloaded from the Department of Natural Resources and Mines (https://water-monitoring.information.qld.gov.au/) under the Creative Commons Attribution 3.0 Australia (CC BY) license. Discharge data from the most downstream gauging station for each major drainage area was grouped into 1° latitudinal blocks (27 stations for the 4 latitudes chosen). The discharge variables were then calculated for each latitude as follows: (1) peak discharge or maximum discharge in a given month across all stations; (2) monthly mean discharge across all stations; (3) cumulative mean for all stations across all stations. Data for each month between 1996 and 2013 was analyzed [12].

Rainfall and air temperature data was obtained from the Bureau of Meteorology for a central coastal station within each latitudinal block with a complete dataset. Mean monthly maximum and minimum air temperatures were used directly. The monthly average daily diurnal air temperature difference was calculated by obtaining the maximum and minimum daily air temperatures and calculating the difference, then averaging this value over the month. Data for each month between 1996 and 2013 was analyzed.

### Data analysis

Data from StrandNet was grouped into 1° latitudinal blocks from the -28° (Queensland–New South Wales border) north to -16° (Cape Tribulation) for each month between January 1996 and December 2013 [12]. Only natural and unknown causes of death were used for this analysis, as anthropogenic causes can be seasonal due to increased activity (eg. Fishing and boating) [12]. The “unknown cause” used as the operating practice for StrandNet was applied when there was no obvious cause of trauma or subsequent analysis done [12].

Strandings were also isolated from bays recognized from the Queensland Spatial Catalogue (http://qldspatial.information.qld.gov.au/catalogue/) under the Department of Natural Resources and Mines CC BY license. Bays were selected as representing an encapsulating body of water including some estuarine and tidal habitats, within which a population may usually reside irrespective of arbitrary coordinates. Standings were mapped using ArcGIS and then overlapped with the Bay layer. For embayment assessment, strandings were only used if they occurred within the defined bay area.

### Model formation

When constructing the model, environmental discharge, air temperature and rain variables were lagged up to 12 months, with a cumulative effect. Time lag one included the environmental factor from time 0 and time -1, time lag two included the environmental factor from time 0, -1 and -2; and so on. A non-cumulative lag effect was also used for this analysis and compared against the cumulative effect.

A 12-month maximum lag time was used as there has historically been links made between marine turtle and dugong deaths occurring within this time frame of extreme weather events [12,20,30]. As seagrass loss after extreme weather events has been noted to be immeditate it is not through to delay the response observed in marine turtle stranding rates [31].

All species of marine turtle to occur within the study area were analyzed individually and collectively as a total count of strandings.

Age classes used for analysis were large immature, adult sized, small immatures, combined small immatures and large immatures, combined large immature and adult sized as well as all age classes together. Models were analyzed where sample sized allowed.

The latitudes with the most strandings (both embayments and whole blocks) were chosen to run the models. These latitudes were -27°, -25°, -23°, -19°.

### Model computation

The models were run as general linear models using R [32] with the bbmle package used to calculate additional information criterion including weights and qAIC values [33,34]. The models were run *a priori* approach due to the complexity and number of possible models [35–37]. Steps followed were similar to those outlined in Bolker et al [35]. Briefly these were specifying the effects, choosing an error distribution, graphically checking variance, fit GLM model to both full model and with each factor.

The strandings data had an excess number of zeros and data was also over-dispersed, so a quasi-Poisson error distribution was used [38].

### Model hypothesis

The hypotheses tested are outlined below:

1. Small minimum air temperature will cause increases in marine turtle stranding rates.
2. Maximum air temperature will not affect marine turtle stranding rates.
3. Increased rainfall will cause increased marine turtle strandings rates 7–9 months later.
4. Increased freshwater discharge will cause increased marine turtle stranding rates 7–9 months later.
5. All environmental factors combined will affect marine turtle stranding rates 7–9 months later.

### Model testing

To begin with, models were run with all variables combined. These models proved non-significant (p > 0.1). After this, each environmental factor was run separately to determine the individual effect. This was done for each age class and species for each latitude chosen. A no effect model was also run for each variable, ageclass and species.

In order to compare models, QAIC weights were calculated using the relative likelihood of the model. This was done following the steps outlined in Bolker [34], briefly the regular model was fit, then the over dispersion parameter was manually extracted to calculate a qAIC value. qAIC is the quasi Akaike Information Criterion (AIC). qAIC weights allow for the selection of a “best approximating model” [36]. This was then used in conjunction with the significance of the variables to determine which model most accurately explained the variance.

Strandings numbers of less than 10 over the 18-year period were excluded due to small sample size as were age class and species with less than 2 turtles per month for the 18-year period.

## Results

### Numbers of animals reported stranded

The number of turtles reported stranded over the 18 years is depicted in **Table 1.**

**Table 1:** Number of marine turtles reported stranded in each latitudinal block. NA represents not analyzed. Bolded latitudes are the recognised hotspots.

Latitude	Number of strandings	
	Whole Block	Embayment
-28	102	NA
**-27**	**5344**	**1391**
-26	1302	NA
**-25**	**1572**	**410**
-24	642	NA
**-23**	**1256**	**158**
-22	228	NA
**-21**	**463**	**NA**
-20	496	NA
**-19**	**1390**	**417**
-18	282	NA
-17	237	NA
-16	411	NA
-15	65	NA
-14	26	NA
-13	1	NA
-12	19	NA
-11	10	NA
-10	7	NA

Upon initial investigation green turtles were the only species which could be analyzed separately due to sample size. For the remaining sections of this study green turtles and the total number of strandings were analyzed and reported.

### Green turtles

#### Rainfall

**Table 2** summarizes the relationship between rainfall and green turtle strandings rates. In brief it shows that within the -19° and -27° blocks strandings decreased as rainfall increased, while the -23 and -25° blocks showed split responses; the majority of age classes showed significant responses within the first 3 months; obvious differences between cumulative and non-cumulative effects of rainfall; different responses time noted with both embayments and whole blocks.

**Table 2:** Model results for green turtles and rainfall. ↑ denotes increased strandings rates with increased rainfall. ↓ denotes decreased stranding rates with increased rainfall. Age class abbreviations: ALL = all turtles, SI = small immature, LI = large immatures, A = adult-sized, ALL IMM = all immature sized animals (small + large), Large = all large turtles (large immatures + adult-sized). The time frame is reported in monthly ranges where responses were noted. The values reported in qAIC are the months with the most significant qAIC value.

Latitude	Age class	Cum—Whole		Cum—bay		Non-Cum Whole		Non-Cum Bay	
		Time Frame	QAIC	Time Frame	QAIC	Time Frame	QAIC	Time Frame	QAIC
**-27**	ALL	2–8↓	12	1↓,2↓,4↓	12	-		-	12,11
	SI	0–12↓	12	1↓,10↓	12	0↓	12,11,10	-	12,11
	LI	1–8↓	12	4-,5↓	12,11,10	1↓,3–4↓	12,11,10	4↓	12,11
	A	-	12	-	12,11,10	0↑	12,11,10	-	12,11
	ALL IMM	0–12↓	12,11,10	1↓,2↓,4↓	12,11	0–1↓	12,11,10	-	12,11
	LARGE	4–5↓	12	4↓	12,11	3↓	12,11,10	-	12,10
**-25**	ALL	0–3↓,7–12↑	10,9,11	0–5↓,9–12↑	2,11,3,12	0–2↓,4–9↑,12↓	8	0–2↓,4–9↑,12↓	8,9
	SI	5–12↑	11,10	8–12↑	11,10,12	3–9↑	7,8	3–9↑	9,8
	LI	0–5↓,9–10↑	2,3,11	0–6↓	12,11,3	0–2↓,5–8↑,11–12↓	8,12	0–2↓,5–8↑,11–12↓	12,11
	A	0–5↓,8–12↑	2,1	0–6↓,9–11↑	2,0,1	0–2↓,5–9↑,12↓	8	0–2↓,5–9↑,12↓	9,7,8
	ALL IMM	0–2↓,6–12↑	10,9,11	0–5↓,9–12↑	11,12,10	0↓,4–9↑	7,8	0↓,4–9↑	8,9
	LARGE	0–5↓,8–12↑	2	0–6↓	2,3,1	0–2↓, 5–9↑,11–12↓	8	0–2↓, 5–9↑,11–12↓	8,9
**-23**	ALL	7–12↑	12,11	0–2↓,7–12↑	12,11,10	6–11↑	12	0↑, 6–9↓	7,8
	SI	2↓,7–12↑	11,12,10	2↓,7–12↑	12,10,11,9	5–9↑	12,1	6–9↓	7,9,8
	LI	8–12↑	12,11,10,9	-	-	1↓,6–10↑	2,12	-	-
	A	0–2↑, 10–12↑	12	0	12,11,10	0↑, 10–12↑	10,12,11	0↓,8↑	11,10,12,9
	ALL IMM	2↓,7–12↑	11,12,10	0–2↓,7–12↑	12,11,10,9	5–9↑	12,11	0↓,4↑,6–9↑	7,9,8
	LARGE	0–1↑,8–12↑	12	0–1↓,11–12↑	12,11,10	0↑,8–11↑	12,10	1↓,8↑	0,11,9
**-19**	ALL	0–7↓,12↓	12	0–6↓,11–12↓	12,2,11	0↓,2↓	12	0↓,2↓	12,11,0,2
	SI	0–6↓	2,3,1,4	0–7↓	2,3,1,4	0–1↓	12,1	0–2↓	0,1,12
	LI	0–12↓	12,5,11	11–12↓	12,11,10	0↓,2↓	2,12	2↓,12↓	12,2,10,11
	A	11–12↓	12	-	12	10↓	10,12,11	10–11↓	10,11,12,9
	ALL IMM	0–7↓	3,2,4	0–7↓	2,3,4	0–2↓	12,11	0–2↓	0,2,11
	LARGE	1↓,5↓,10–12↓	12	11–12↓	12	2↓,10↓	12,10	10↓,12↓	12,10,11

QAIC’s for all groups assessed were different and no patterns were observed (**Table 2**). In most cases, the QAICs corresponded with significant responses, with an exception for the age classes which did not produce a significant relationship.

#### Cumulative mean and mean freshwater discharge

Similar patterns in response for cumulative mean discharge and mean discharge and stranding rates were noted **(Tables 3 and 4)**. There were different lag response times but the patterns remained the same. As such, analysis for both measures are discussed together. The only exception was all green turtles within the -25° block for cumulative lag effects of cumulative mean discharge in the whole block did not show the initial decrease that was observed in the mean discharge **(Tables 3 and 4)**.

**Table 3:** Model results for green turtles and cumulative mean discharge. ↑ denotes increased strandings rates with increased discharge. ↓ denotes decreased stranding rates with increased discharge. Age class abbreviations: ALL = all turtles, SI = small immature, LI = large immatures, A = adult-sized, ALL IMM = all immature sized animals (small + large), Large = all large turtles (large immatures + adult-sized). The time frame is reported in monthly ranges where responses were noted. The values reported in qAIC are the months with the most significant qAIC value.

Latitude	Age class	Cum—Whole		Cum—bay		Non-Cum Whole		Non-Cum Bay	
		Time Frame	QAIC	Time Frame	QAIC	Time Frame	QAIC	Time Frame	QAIC
-27	ALL	7–12↑	12	8–12 ↑	12	1↑,6–9↑	8	1↑,5↑,8–10↑	12
	SI	5–12↑	12	7–12↑	12,11	6–9↑,11↑	7,8	6↑,8–11↑	8
	LI	11–12↓	12,11	12↓	12,11	-	11,12	-	11,12
	A	9–10↑	12,11	9–12↑	12,11	7–9↑	12,11.10	8–9↑	12,11
	ALL IMM	7–12↑	12	8–11↑	12,11	5–8↑	12,8,7	6↑,8–9↑	12,8
	LARGE	0↓	12	-	12,11	1↓,8↑	12,8	5↑	12,11
-25	ALL	6–12↑	10,11,9	3↓,8–12↑	11,10	5–9↑	8	6–10↑	8
	SI	4–12↑	11,10	7–12↑	11,12,10,9	3–10↑	7,8	6–11↑	8,7
	LI	0–4↓,8–12↑	12,11	1–4↓	11,12,10	0–2↓,5–9↑	8,7	1↓,6–9↑	12,11,8
	A	0–3↓,8–12↑	10,12,11	0–2↓,8–12↑	11,10,12	0–1↓,6–9↑	8	0↓,6–9↑	7,8
	ALL IMM	6–12↑	10,11	7–12↑	11,12,10	4–9↑	7	6–10↑	8,9
	LARGE	0–4↓,8–12↑	10,11,12	0–4↓,8–12↑	11,12,10	0–2↓,5–9↑	8	0↓,6–9↑	8,7
-23	ALL	3–12↑	11,12	4–12↑	11,10,12	3–11↑	7	3–11↑	7
	SI	4–12↑	11,10	3–12↑	11,10,12,9	3–11↑	7	3–11↑	6,7
	LI	7–12↑	11,12,10,9	-	-	5–11↑	8,7	-	-
	A	0–12↑	12	7–12↑	11,10,12,9	0–1↑,6–12↑	10,9	6–8↑	7,8
	ALL IMM	4–12↑	11,10	3–12↑	11,10,12	3–11↑	7	3–11↑	6,7
	LARGE	0–12↑	12	7–12↑	11,12,10	0↑,5–12↑	8,10,9	7–11↑	8,7,10
-19	ALL	5–12↑	10,9,11	4–12↑	9,10,11	3–8↑	7,8,6,5	3–8↑	5,6
	SI	5–12↑	9,10	5–12↑	9,10,11,8	3–8↑	6,5,7	3–8↑	5,6
	LI	6–12↑	11,10,12,9	6–12↑	9,10,8,11	3↑,6–9↑	8,9	3–4↑,6↑	3,8,6,4
	A	-	11,12,10	4–12↑	10,11,9,8	3↑	3	3–5↑	3
	ALL IMM	5–12↑	10,9	5–12↑	9,10,11,8	3–9↑	6,7,5	3–8↑	5,6
	LARGE	8–12↑	11,10,12,9	4–12↑	10,9,8,11,12	3↑	3	3–5↑	3

**Table 4:** Model results for green turtles and mean discharge. ↑ denotes increased strandings rates with increased discharge. ↓ denotes decreased stranding rates with increased discharge. Age class abbreviations: ALL = all turtles, SI = small immature, LI = large immatures, A = adult-sized, ALL IMM = all immature sized animals (small + large), Large = all large turtles (large immatures + adult-sized). The time frame is reported in monthly ranges where responses were noted. The values reported in qAIC are the months with the most significant qAIC value.

Latitude	Age class	Cum—Whole		Cum—bay		Non-Cum Whole		Non-Cum Bay	
		Time Frame	QAIC	Time Frame	QAIC	Time Frame	QAIC	Time Frame	QAIC
**-27**	ALL	7–1 2↑	12	8–12↑	12	1↑,6–9↑	8	1↑,5↑,8–10↑	12
	SI	5–12↑	12	7–12↑	12,11	6–9 ↑,11↑	8	6↑,8–11↑	8
	LI	11–12↓	12,11	12↓	12,11	-	12,11	-	11,12
	A	9–10↑	12,11	9–12↑	12,11	7–9↑	12	8–9↑	12,11
	ALL IMM	7–12↑	12	8–12↑	12,11	5–8↑	12,8	6↑,8–9↑	12,8
	LARGE	0↓	12	-	12	1↓,8–9↑	12,8	5↑	12,11
**-25**	ALL	2↓,7–12↑	10,11	2–3↓,8–12↑	11,10	5–9↑	8	6–10↑	8
	SI	5–12↑	11,10	7–12↑	11,12,10,9	3–10↑	7,8	6–11↑	8
	LI	0–4↓,8–11↑	12,11	1–4↓	11,12,10	0–2↓,5–9↑	8,7	1↓,6–9↑	12,11
	A	0–3↓,8–12↑	10,12,11	0–2↓,8–12↑	11,10,12	0–1↓,6–9↑	8	0↓,7–9↑	7,8,9
	ALL IMM	6–12↑	10,11	7–12↑	11,12,10	4–9↑	7	6–10↑	8,9
	LARGE	0–4↓,8–12↑	10,11,12	0–4↓,8–12↑	11,12,10	0–2↓,5–9↑	8	0↓,6–9↑	8,7
**-23**	ALL	3–12↑	11,12	4–12↑	11,10,12	3–11↑	7	3–11↑	7
	SI	4–12↑	11,10	3–12↑	11,10,12,9	3–11↑	7	3–11↑	6,7
	LI	7–12↑	11,12,10,9	-	-	5–11↑	8,7	-	-
	A	0–12↑	12	7–12↑	11,10,12,9,8	0–1↑,6–12↑	10,9	7–8↑	7,8
	ALL IMM	4–12↑	11,10	3–12↑	11,10,12	3–11↑	7	3–11↑	6,7
	LARGE	0–12↑	12	7–12↑	11,12,10	0↑,5–12↑	8,10	7–11↑	8,7,10
**-19**	ALL	5–12↑	10,9,11	4–12↑	9,10,11	3–8↑	7,8,6,5	3–8↑	5,6
	SI	5–12↑	9,10	5–12↑	9,10,11,8	3–8↑	6,5,7	3–8↑	5,6
	LI	6–12↑	11,10,12,9	6–11↑	9,10,8,11	3–4↑,6–9↑	8,9	3–4↑,6↑	3,8,6,4
	A	-	11,12,10,9	4–12↑	10,11,9,8	3↑	3	3–5↑	3
	ALL IMM	5–12↑	10,9	5–12↑	9,10,11,8	3–9↑	6,7,5	3–8↑	5,6
	LARGE	8–12↑	11,10,12,9	4–12↑	10,9,8,11	3↑	3	3–5↑	3

Differences in the examined latitudinal blocks were observed **(Tables 3 and 4)**.

Within each examined latitudinal block, there were no observed pattern as to which age class was the first to show significant responses **(Tables 3 and 4)**.

These patterns did not change when comparing embayment’s with whole blocks but the lag time may be extended when examining strandings within the embayment compared to whole block strandings **(Tables 3 and 4)**.

All examined latitudinal blocks for non-cumulative lagged effects responded similarly to cumulative effects, with non-cumulative showing responses first **(Tables 3 and 4)**.

QAIC’s for all groups assessed were different and no patterns were observed **(Tables 3 and 4)**. In most cases, the QAICs corresponded with significant model responses, with an exception for the age classes which did not produce a significant relationship.

#### Peak discharge

**Table 5** summarizes the relationships between green turtle stranding numbers and peak discharge. In brief, large immatures and large turtles in the -27° block showed no significant response; in the -19°, -23° and -27° degree blocks, as peak discharge increased so did green turtle stranding rates, as a comparison, in the -25° block showed a split response with strandings decreasing with increased discharge over the first 5 months, which then switched to increased strandings with increasing peak discharge.

**Table 5:** Model results for green turtles and peak discharge. ↑ denotes increased strandings rates with increased discharge. ↓ denotes decreased stranding rates with increased discharge. Age class abbreviations: ALL = all turtles, SI = small immature, LI = large immatures, A = adult-sized, ALL IMM = all immature sized animals (small + large), Large = all large turtles (large immatures + adult-sized). The time frame is reported in monthly ranges where responses were noted. The values reported in qAIC are the months with the most significant qAIC value.

Latitude	Age class	Cum—Whole		Cum—bay		Non-Cum Whole		Non-Cum Bay	
		Time Frame	QAIC	Time Frame	QAIC	Time Frame	QAIC	Time Frame	QAIC
**-27**	ALL	7–12↑	12	9–12↑	12	6–9↑	8	8–10↑	12,10
	SI	6–12↑	12	8–12↑	12,11	6–9↑,11↑	11,8,12	6↑,8–11↑	8
	LI	-	12,11	-	12,11	-	11,12	-	12,11
	A	9–12↑	12,11	8–12↑	12,11	9–10↑	12,11,10	8–9↑	12,11
	ALL IMM	7–12↑	12	10–12↑	12,11	6–8↑	12	8↑,10↑	12,10
	LARGE	-	12	-	12	8↑	12,9,8	-	12,11
**-25**	ALL	2↓,7–12↑	10,11	3↓,8–12↑	11,10	6–9↑	8	6–10↑	8
	SI	5–12↑	11,10	7–12↑	11,12,10,9	5–10↑	7	6–10↑	8,7
	LI	1–5↓,8–12↑	11,12	1–4↓,9–11↑	11,12,10	1–2↓,5–9↑	8	1↓,3↓,5↑,7–9↑	12,11,8
	A	0–5↓,8–12↑	10,11,12	0↓,9–12↑	11,10,12	0–1↓,6–9↑	8	0↓,7–9↑	8,9,7
	ALL IMM	6–12↑	10,11	8–12↑	11,12,10	5–9↑	7,8	7–9	8
	LARGE	0–5↓,8–12↑	10,11,12	0–5↓,9–12↑	11,12,10	0–2↓,6–9↑	8	0↓,7–9↑	8
**-23**	ALL	4–12↑	11,12	5–12↑	11,10,12	3–11↑	7	3–11↑	7
	SI	4–12↑	11,10	4–12↑	11,10,12,9	3–11↑	7	3–11↑	6,7
	LI	6–12↑	11,12,10,9	-	-	3↑,5–11↑	7,8	-	-
	A	0–12↑	12	7–12↑	11,10,12,9	0–1↑,6–12↑	12,10,9,8	6–8↑,10↑	7,8,10
	ALL IMM	4–12↑	11,10	4–12↑	11,10,12,9	3–11↑	7	3–11↑	6,7
	LARGE	0–12↑	12	7–12↑	11,12,10	0↑,5–12↑	8,7	6–11↑	7,8,10
**-19**	ALL	5–12↑	10,9	4–12↑	9,10	3–8↑	3	3–8↑	3,5
	SI	5–12↑	9,10	5–12↑	9,10,11	4–8↑	5,6	4–8↑	5
	LI	6–12↑	10,11,9	5–12↑	9,8,10,11	3–4↑,6↑, 8–9↑	8,3,9	3–4↑,6↑	3,6,4,8
	A	4–11↑	10,11,9,12	3–12↑	10,9,8	3↑	3	3–5↑	3
	ALL IMM	5–12↑	10,9	5–12↑	10,9,11	3–9↑	6,5,7	3–8↑	5,6
	LARGE	8412↑	10,11,9	3–12↑	9,10,8,	3↑	3	3–5↑	3

**Table 5** also displays that within each examined latitudinal block, most age classes showed a significant stranding response to peak discharge, however, there was no observed pattern as to which age class was the first to show significant responses; all examined latitudinal blocks for non-cumulative lagged effects of peak discharge, responded similarly to cumulative effects, with non-cumulative showing responses first.

These patterns did not change when comparing embayments with whole blocks but the lag time may be extended when examining strandings within the embayment compared to whole block green turtle strandings **(Table 5)**.

QAIC’s for all groups assessed were different and no patterns were observed **(Table 5)**. In most cases, the QAICs corresponded with significant responses, with an exception for the age classes which did not produce a significant relationship.

#### Monthly mean maximum air temperature

**Table 6** summarizes the relationship between monthly mean maximum air temperature and green turtle stranding rates. In brief it shows that in most cases, as monthly mean maximum air temperatures increased the green turtle stranding rate decreased; there was a significant response noted within the first 4 months; there were very obvious differences between cumulative and non-cumulative effects of monthly mean maximum air temperature, with non-cumulative effects more likely to produce split responses; and that there were similar stranding response times noted with both embayments and the whole blocks for monthly mean maximum air temperature.

**Table 6:** Model results for green turtles and monthly mean maximum air temperature. ↑ denotes increased strandings rates with increased mean maximum air temperature. ↓ denotes decreased stranding rates with increased mean maximum air temperature. Age class abbreviations: ALL = all turtles, SI = small immature, LI = large immatures, A = adult-sized, ALL IMM = all immature sized animals (small + large), Large = all large turtles (large immatures + adult-sized). The time frame is reported in monthly ranges where responses were noted. The values reported in qAIC are the months with the most significant qAIC value.

Latitude	Age class	Cum—Whole		Cum—bay		Non-Cum Whole		Non-Cum Bay	
		Time Frame	QAIC	Time Frame	QAIC	Time Frame	QAIC	Time Frame	QAIC
**-27**	ALL	0–9 ↓	6	0–9↓	6	0–4↓,6–10↑,12↓	8,9	0–4↓,6–10↑,12↓	8
	SI	0–8↓,12 ↓	4,12	0–9↓,11–12↓	12,6	0–4↓,6–9↑,12↓	12,8	0–4↓,6–10↑,12↓	8
	LI	1–8 ↓	6	2–8↓	12,11	1–5↓,7–11↑	9	1–4↓,7–10↑	10,8,12
	A	1–9 ↓	6	0–7↓	12,11	1–5↓,7–11↑	9	0–3↓,6–9↑	12,8
	ALL IMM	0–9 ↓	5	0–9↓,12↓	7,6	0–4↓,6–10↑	8	0–4↓,6–10↑,12↓	8,7
	LARGE	1–9 ↓	6,5	0–8↓	12,11	1–5↓,7–11↑	9	0–4↓,6–10↑	8,9
**-25**	ALL	1–8↓,11↑	5,4	2–8↓,10–12↑	11,5,12,6	0–4↓, 6–10↑	8	1–5↓, 7–11↑	9
	SI	0–5↓,10–11↑	10,3,11	3–7↓,11↑	11,12,5	0–3↓, 5–10↑,12↓	7	2–4↓,7–10↑	9,10,8
	LI	2–8↓	5,4	2–8↓,11–12↑	11,12	1–4↓, 6–10↑	3,9,8	1–4↓, 7–11↑	9,11,10
	A	2–9↓	6,5	0↑,3–8↓,11–12↑	11,12,5	0–5↓, 7–11↑	9	0↑,2–5↓, 7–11↑	9,8
	ALL IMM	0–7↓,10–11↑	4,3	2–8↓,11–12↑	11,12,5,6	0–3↓, 6–10↑	7,8	1–4↓, 7–11↑	9,8
	LARGE	2–8↓	5,6	2–8↑↓,11–12↑	11,12,5,6	1–5↓, 7–11↑	3,9	1–5↓, 7–11↑	9
**-23**	ALL	0–12↓	6,5,7	0–12↓	4,3,12,5	1–4↓, 8–9↑	3,2	0–3↓, 6–8↑,12↓	2,3,1,12
	SI	0–7↓,11–12↓	3,4	0–12↓	4,12,3,5	0–3↓, 6–8↑,12↓	1,2,12	0–3↓, 7–8↑,12↓	2,1,3
	LI	1–11↓	5,4,6	-	-	1–4↓,7–9↑	2,3,8,12	-	-
	A	0↑↓,5–12↓	9,8,10	-	12,11,10	0↑, 3–7↓,9–12↑	5	-	12,11,10
	ALL IMM	0–8↓, 10–12↓	3,4	0–12↓	4,3,5,12	0–3↓, 6–8↑,12↓	2,1	0–3↓, 7–8↑,12↓	2,3,1,12
	LARGE	4–12↓	8,9	2–5↓	12,11,10	2–6↓,9–11↑	5,4	1–3↓	12,2,11,10
**-19**	ALL	0–12↓	12	0–12↓	12	0–3↓, 6–7↑,11–12↓	12	0–2↓, 6–7↑,11–12↓	12
	SI	0–12↓	12	0–12↓	12	0–3↓, 6–8↑,11–12↓	1	0–3↓, 6–7↑,11–12↓	1
	LI	0–12↓	12	0–6↓,10–12↓	12	0–3↓,12↓	12	0–3↓,7↑,12↓	12,1,0,11
	A	0–3↓,11–12↓	12	0–4↓,11–12↓	12	0–1↓, 11–12↓	12	0–1↓, 6↑,11–12↓	12
	ALL IMM	0–12↓	12	0–12↓	12	0–3↓, 6–8↑,11–12↓	1	0–3↓, 6–7↑,11–12↓	1,12
	LARGE	0–5↓,11–12↓	12	0–5↓,10–12↓	12	0–1↓,12↓	12	0–1↓,6↑,12↓	12

QAIC’s for all groups assessed were different and no patterns were observed **(Table 6)**. In most cases, the QAICs corresponded with significant responses, with an exception for the age classes which did not produce a significant relationship.

#### Monthly mean minimum air temperature

**Table 7** summarizes the relationship between monthly mean minimum air temperature and green turtle stranding rates. In brief, it shows in most cases, as monthly mean minimum air temperatures increased the stranding rate decreased; there were very obvious differences between cumulative and non-cumulative effects, with non-cumulative effects resulted in split responses; in most cases there was a significant green turtle strandings response noted within the first 3 months of the mean minimum air temperature recorded; there were similar responses time noted with both embayments and whole blocks.

**Table 7:** Model results for green turtles and monthly mean minimum air temperature. ↑ denotes increased strandings rates with increased mean minimum air temperature. ↓ denotes decreased stranding rates with increased mean minimum air temperature. Age class abbreviations: ALL = all turtles, SI = small immature, LI = large immatures, A = adult-sized, ALL IMM = all immature sized animals (small + large), Large = all large turtles (large immatures + adult-sized). The time frame is reported in monthly ranges where responses were noted. The values reported in qAIC are the months with the most significant qAIC value.

Latitude	Age class	Cum—Whole		Cum—bay		Non-Cum Whole		Non-Cum Bay	
		Time Frame	QAIC	Time Frame	QAIC	Time Frame	QAIC	Time Frame	QAIC
**-27**	ALL	0–9 ↓	6	0–9↓	6	0–4↓,6–11↑,12↓	8	0–4↓,6–10↑,12↓	8,9
	SI	0–8↓,12 ↓	4,12	0–8↓	7,6	0–3↓,6–10↑,12↓	8	0–4↓,6–10↑,12↓	8,9
	LI	1–9 ↓	6	1–8↓	12,11	1–4↓,7–11↑	9	0–4↓,8–9↑	9,12,10
	A	1–9 ↓	6	0–6↓	12,11	1–5↓,7–11↑	9	0–3↓,5–9↑,11–12↓	12
	ALL IMM	0–9↓,12 ↓	4,5	0–8↓	7,6	0–4↓,6–10↑,12↓	8	0–4↓,6–10↑,12↓	8,9
	LARGE	1–9 ↓	6	0–8↓	12,11	1–5↑,7–11↓	9	0–4↓,6–9↑,12↓	9,8
**-25**	ALL	0–8↓,12↓	4,5	1–10↓	5,6,4	0–4↓, 6–10↑, 12↓	8,2	1–5↓,7–11↑	9,2
	SI	0–6↓,12↓	12,2,3	1–10↓,12↓	5,6,4,7	0–3↓, 5–8↑,11–12↓	12	1–4↓, 7–10↑	9,2
	LI	1–8↓	4,5	1–9↓	12,7,11	1–4↓, 6–10↑,12↓	8,9	1–4↓, 6–10↑	9,8
	A	1–9↓	5,6	1–10↓	5,6,7,4	1–5↓, 7–11↑	9	1–5↓, 6–10↑	9,8
	ALL IMM	0–7↓,102↓	3,4	1–10↓,12↓	5,4,6,7	0–3↓, 6–10↑,12↓	7,2	1–4↓, 6–10↑	9,8
	LARGE	1–9↓	5	1–10↓	5,6,4,7	1–4↓,6–11↑	9	1–5↓,7–11↑	9,8
**-23**	ALL	0–12↓	5,4,6	0–8↓,10–12↓	12,3,2,4	0–4↓, 7–9↑	2	0–3↓, 6–8↑,12↓	2,1,7
	SI	0–7↓,11–12↓	3,2	0–7↓,10–12↓	3,2,4,12	0–3↓, 6–8↑,12↓	1	0–3↓, 6–9↑,12↓	2,1,7
	LI	0–12↓	4,12,5,6	-	-	0–4↓,6–9↑,12↓	2,8,12	-	-
	A	0↑,5–10↓	8,9	12↓	12,11,10	0↑, 3–6↓,9–12↑	11,5,10	2↓	12,11,10
	ALL IMM	0–7↓,11–12↓	3,2	0–8↓, 10–12↓	3,2,4,12	0–3↓, 6–9↑,12↓	1,2	0–3↓, 6–9↑	2,1,7
	LARGE	3–11↓	8,9,7	1–5↓,11–12↓	12,11,10	2–6↓,8–112↑	10,11	1–3↓	12,2,11,10
**-19**	ALL	0–5↓,12↓	12	0–5↓,12↓	12	0–2↓, 6–8↑,11–12↓	12	0–2↓, 5–8↑,11–12↓	12,0
	SI	0–6↓,12↓	2,3	0–6↓,12↓	2,1,3	0–3↓, 5–9↑,11–12↓	1,0,7	0–2↓, 5–8↑,11–12↓	0,1,7
	LI	0–6↓,11–12↓	12	0–4↓,12↓	12	0–3↓,7–8↑,12↓	12,8,2	0–2↓,6–7↑,12↓	12,7,1
	A	0–3↓,11–12↓	12	0–2↓,12↓	12	0↓, 11–12↓	12	0–1↓, 5–6↑,11–12↓	12,11,0
	ALL IMM	0–6↓,12↓	2, 3	0–5↓,12↓	2,1,12,3	0–3↓, 5–9↑,12↓	1,7	0–3↓, 5–8↑,11–12↓	0,1,12
	LARGE	0–3↓,11–12↓	12	0–3↓,12↓	12	0–1↓,12↓	12	0–1↓,5–7↑,11–12↓	12

QAIC’s for all groups assessed were different and no patterns were observed **(Table 7)**. In most cases, the QAICs corresponded with significant responses, with an exception for the age classes which did not produce a significant relationship.

#### Monthly average daily diurnal air temperature difference

**Table 8** summarizes the relationship between monthly average daily diurnal air temperature difference and green turtle stranding rates. In brief it shows very obvious differences between cumulative and non-cumulative effects of monthly average daily diurnal air temperature differences; Non-cumulative effects resulted in split responses whereas in most cases the cumulative effects resulted in decreased stranding rate with increased mean minimum air temperature; in most cases there was a significant response noted within the first 3 months of monthly average daily diurnal air temperature difference being recorded; similar response times were noted with both embayments and whole blocks.

**Table 8:** Model results for green turtles and monthly average daily diurnal air temperature difference. ↑ denotes increased strandings rates with increased average daily diurnal air temperature difference. ↓ denotes decreased stranding rates with increased average daily diurnal air temperature difference. Age class abbreviations: ALL = all turtles, SI = small immature, LI = large immatures, A = adult-sized, ALL IMM = all immature sized animals (small + large), Large = all large turtles (large immatures + adult-sized). The time frame is reported in monthly ranges where responses were noted. The values reported in qAIC are the months with the most significant qAIC value.

Latitude	Age class	Cum—Whole		Cum—bay		Non-Cum Whole		Non-Cum Bay	
		Time Frame	QAIC	Time Frame	QAIC	Time Frame	QAIC	Time Frame	QAIC
**-27**	ALL	0–8 ↑	6	0–8↑	12	0–4↑,6–10↓	8	0–3↑,6–10↓,12↑	8,9
	SI	0–6 ↑	12,11	0–6↑	12,11	0–3↑,6–10↓,12↑	8	0–3↑,6–10↓	8,9
	LI	1–9 ↑	6	0–8↑	12,11,10	1–4↑,7–11↓	9	0–3↑,8–9↓	9,12,11
	A	1–8 ↑	5,4	0–5↑	12,11	1–4↑,7–10↓	9	0–2↑,5–9↓,11–12↑	12
	ALL IMM	0–7 ↑	4	0–7↑	12,11	0–4↑,6–10↓,12↑	8	0–3↑,6–10↓	9,8
	LARGE	1–8 ↑	6	0–8↑	12,11	1–4↑,7–11↓	9	0–3↑,6–9↓,12↑	12
**-25**	ALL	0–8↑,12↑	5,4	0–12↑	5,4,3,2	0–3↑, 6–9↓,12↑	2,1	0–4↑, 7–10↓	2
	SI	0–7↑,11–12↑	10,3,11	0–12↑	4,5,3,2	0–2↑,6–8↓,11–12↑	12	0–4↑,9↓,12↑	12,0
	LI	0–7↑	5,4	0–9↑,12↑	12,11,4	0–3↑,6–9↓,12↑	8,7	0–3↑,6–9↓,12↑	12,9
	A	0–8↑	6,5	0–12↑	5,4,6,3	0–4↑, 6–10↓,12↑	8,9,2	0–4↑, 7–10↓	2
	ALL IMM	0–7↑,12↑	4,3	0–12↑	4,3,5,2	0–3↑, 5–9↓,11–12↑	12,0	0–4↑, 6–10↓,12↑	12,1
	LARGE	0–8↓	5,6	0–12↑	5,4,3	0–4↑,6–10↓,12↑	8	0–4↑,7–10↓,12↑	2
**-23**	ALL	0–3↑	12,11,10	0–3↑,	12,11,2,10	0↑, 5–9↓	9,8	0↑, 5–7↓	5,6,12,9
	SI	0–2↑	12,11,10,9	0–1↑,8–12↓	12,11,10,9	0↑,5↓	5	0↑,5–7↓,9↓	5,9,7,6
	LI	0–4↑	12,11,10	-	-	0–1↑,6↓	12,11,10	-	-
	A	11–12↓	12	2–4↑	12,11,10	3–4↑,6↓,8–11↓	10,9,12,8	2↑	2,11,12,10
	ALL IMM	0–3↑	12,2,11	0–2↑, 9–12↓	12,11,10	0↑, 5↓	5	0↑, 5–7↓,9↓	6,7,5
	LARGE	3–4↑	12	2–4↑	12,11,10	6↓,9–10↓	10,9,12	2↑	11,12,10,2
**-19**	ALL	0–3↑, 8–12↓	10	0–3↑, 7–12↓	10,9	0–2↑, 5–8↓	7,8,6	0↑, 4–8↓	7,8,6
	SI	0–3↑,7–12↓	10,11	0–3↑,7–12↓	10,11,9	0–2↑,4–9↓	7,6	0–1↑,5–9↓	7,6
	LI	-	11,12,10	8–10↓	9,10,11,8	6↓,8↓	8,6	5–7↓	6,7,5,8
	A	-	12,9,8	7–10↓	9,8,10	-	12,11	3–5↓	5,11,7,12
	ALL IMM	0–3↑, 7–12↓	10,11	0–3↑, 7–12↓	10,11,9	0–2↑, 5–9↓	6,7	0–2↑, 4–9↓	7,6,8
	LARGE	-	10,9,11	7–10↓	9,8,10	-	12,8,7,11	4–7↓,11↑	5,11

The exception was the -19° block, where significant response times were varied for small immatures, immature and all green turtles and adults and large turtles within the -19° block did not display a significant response **(Table 8)**.

QAIC’s for all groups assessed were different and no patterns were observed. In most cases, the QAICs corresponded with significant responses, with an exception for the age classes which did not produce significant relationship **(Table 8)**.

### All marine turtle strandings

#### Rainfall

**Table 9** summarizes the relationship between rainfall and marine turtle stranding rates. In brief it shows that when comparing rainfall across all blocks, there were different patterns noted for each block; cumulative effects within the -27° block stranding rates decreased as rainfall increased; non-cumulative effects within the -27° block showed mixed results; within each examined latitudinal block, there were similar stranding response times noted for embayments and the whole blocks; there were very obvious differences between cumulative and non-cumulative effects of rainfall on all turtle stranding rates (**Table 9**).

**Table 9:** Model results for all turtles and rainfall. ↑ denotes increased strandings rates with increased rainfall. ↓ denotes decreased stranding rates with increased rainfall. Age class abbreviations: ALL = all turtles, SI = small immature, LI = large immatures, A = adult-sized, ALL IMM = all immature sized animals (small + large), Large = all large turtles (large immatures + adult-sized). The time frame is reported in monthly ranges where responses were noted. The values reported in qAIC are the months with the most significant qAIC value.

Latitude	Age class	Cum-Whole		Cum-Bay		Non Cum-Whole		Non Cum-Bay	
		Time Frame	QAIC	Time Frame	QAIC	Time Frame	QAIC	Time Frame	QAIC
**-27**	ALL	4↓	12,11	-	12,11,10	-	12,11	-	12,11
	SI	0–6↓	12,11	0–4↓	12,11,10	0–4↓	12,11	0	12,11,10
	LI	1–7↓	12,11,10	4↓	12,11,10	1,3–4	12,11,10	5↑,8↑	12,11,10,
	A	1↓	12,11	-	12,11	1	12,11,10	5↑	12,11
	ALL IMM	1–8↓	12,11	0–4↓	12,11,10	0–1	12,11	0↓	12,11,10
	LARGE	-	12,11	2↓	12,11	-	12,11,10	5↑	12,11,10
**-25**	ALL	0–4↓,7–12↑	10,11	0–5↓,9–12↑	2,11,3,12	0–2↓,4–9↑,12↓	8	0–2↓,6–9↑	8
	SI	5–12↑	11,10	8–12↑	11,10,12,9	3–9↓	7,8	0↓,5–10↑	8,7,9
	LI	0–5↓	2,3	0–6↓	12,11,3,2	0–2↓,5–8↑,11–12↓	12	0–3↓,7–8↑,12↓	12,11,8
	A	0–5↓,9–12↑	2,1,12	0–6↓,9–11↑	2,0,1,3	0–2↓,6–9↑,12↓	8,7	0–2↓,6–9↑	7,9,8
	ALL IMM	0–2↓,7–12↑	10,11,9	0–5↓,9–12↑	11,12,10,9	0↓,2↓,4–9↑	8,7	0–2↓,5–9↑,12↓	8
	LARGE	0–5↓,9–11↑	2	0–6↓	2,3,1	0–2↓,5–9↑,12↓	8	0–2↓,6–9↑,12↓	8,7,9
**-23**	ALL	7–12↑	12,11	0–2↓,7–12↑	12,11,10	6–10↑	8,9	0↓,6–9↑	7,0,8
	SI	0–3↓,7–12↑	11,12,10	0↓,2↓,7–12↑	12,11,10	5–9↑	8,7	0↓,4↑,6–9↑	7
	LI	-	-	-	-	-	-	-	-
	A	0–2↑,10–12↑	12	0–1↓	12,11,10	9–12↑	11,10,12	0↓,8↑	11,10,12,9
	ALL IMM	0–3↓,7–12↑	11,12,10	0–2↓,7–12↑	12,11,10	5–9↑	8	0↓,4↑,6–9↑	7
	LARGE	0↑,8–12↑	12	0–2↓,11–12↑	12,11,10	8–11↑	10,11	0↓,8↑	0,11,10,9
**-19**	ALL	0–7↓,12↓	12	0–6↓,12↓	12,2,11	0↓,2↓	12	0↓,2↓	12
	SI	0–6↓	2,3,4	0–7↓	2,1,3,4,5	0–1↓	12,11	0–1↓	0,1
	LI	0↓,2–7↓,10–12↓	12,5,11,4	-	12,11	0↓,2↓	12,10	12↓	12,10,11
	A	11–12↓	12	11–12↓	12,11	10↓	10,12,11	10↓	10,12,11
	ALL IMM	0–7↓	4,3,2	0–7↓	2,3,12	0–2↓	12,12,10	0–2↓	10,12,11
	LARGE	5↓,11–12↓	12	12↓	12,11	2↓		10↓,12↓	12,0,11

QAIC’s for all groups assessed were different and no patterns were observed (**Table 9**). In most cases, the QAICs corresponded with significant responses, with an exception for the age classes which did not produce a significant relationship.

#### Cumulative mean and mean discharge

**Tables 10 and 11** summarizes the relationship between cumulative mean, mean discharge and all marine turtle stranding rates. In brief, it shows in most cases, as cumulative mean discharge increased, the stranding rate for all turtles also increased; each examined latitudinal block, there were similar stranding response times noted for embayments and the whole blocks in respect to discharge; within each examined latitudinal block, there were also similar response times for cumulative effect vs non-cumulative effect of discharge.

**Table 10:** Model results for all turtles and cumulative mean discharge. ↑ denotes increased strandings rates with increased discharge. ↓ denotes decreased stranding rates with increased discharge. Age class abbreviations: ALL = all turtles, SI = small immature, LI = large immatures, A = adult-sized, ALL IMM = all immature sized animals (small + large), Large = all large turtles (large immatures + adult-sized). The time frame is reported in monthly ranges where responses were noted. The values reported in qAIC are the months with the most significant qAIC value.

Latitude	Age class	Cum-Whole		Cum-Bay		Non Cum-Whole		Non Cum-Bay	
		Time Frame	QAIC	Time Frame	QAIC	Time Frame	QAIC	Time Frame	QAIC
**-27**	ALL	8–12↑	12,11	9–12↑	12	1↑,7–9↑	8	1↑,7–10↑	8,10
	SI	5–12↑	12,11	6–12↑	12,11	6–12↑	7,11,8	1↑,5–11↑	8,11
	LI	-	12,11,10	-	12,11,10	-	12,11,10	-	12,11,10
	A	8–12↑	12,11,10	8–12↑	12,11,10	7–9↑	8	10-Jul	8,9
	ALL IMM	7–12↑	12,11	8–12↑	12,11,10	6–11↑	7,8,11	1↑,7–11↑	11,8,12,10
	LARGE	9–11↑	12,11,10	9–12↑	12,11,10	7–9↑	8	7–10↑	12,10
**-25**	ALL	2↓,7–12↑	10,11	3↓,8–12↑	11,10,12	5–10↑	8	6–10↑	8
	SI	4–12↑	11,10,12	7–12↑	11,12,10	3–10↑	8,7	5–11↑	8,7,10,9
	LI	0–4↓,8–11↑	12,11,10	1–4↓	11,12,10	0–2↓,5–9↑	8,7,12	1↓,3↓,7–9↑	11,12,8
	A	0–3↓,8–12↑	12,10,11	0–2↓,8–12↑	11,10,12	0–1↓,6–9↑	8	0↓,6–10↑	7,8,9
	ALL IMM	6–12↑	10,11	7–12↑	11,12,10	4–10↑	7,8	5–10↑	8
	LARGE	0–4↓,8–12↑	12,11,10	0–4↓,8–12↑	11,12,10	0–2↓,5–9↑	8	0–1↓,6–9↑	8,7
**-23**	ALL	4–12↑	11,12	3–12↑	11,10,12	3–11↑	7	3–11↑	7,6
	SI	4–12↑	11,10	3–12↑	10,11,9,12	3–11↑	7	3–11↑	6,7
	LI	-	-	-	-	-	-	-	-
	A	0–12↑	12	7–12↑	11,12,10	0–1↑,6–12↑	10,9	7–11↑	7,8,10
	ALL IMM	4–12↑	11,10	4–12↑	11,10,12	3–11↑	7	3–11↑	6,8
	LARGE	0–12↑	12	7–12↑	11,12,10	0↑,5–12↑	10,8,9	6–11↑	8,7,10
**-19**	ALL	5–12↑	10,11	4–12↑	10,9,11	3–9↑	7,8	3–8↑	5
	SI	5–12↑	10,9,11	5–12↑	10,9,11,8	3–9↑	6,7,5	3–8↑	5,6
	LI	6–12↑	10,11,9,12	4–12↑	9,10,8,11	3–4↑,6↑,8–9↑	8,9	3–4↑	4,3,8,6
	A	-	11,12,10	4–12↑	10,11,9,8	3↑	3	3–5↑	3,5
	ALL IMM	5–12↑	10,9	4–12↑	9,10,11,8	3–9↑	6,7,8	3–8↑	5,6
	LARGE	8–12↑	11,10,12	4–12↑	10,9,11,8	3↑	3	3–5↑	3,5,4

**Table 11:** Model results for all turtles and mean discharge. ↑ denotes increased strandings rates with increased discharge. ↓ denotes decreased stranding rates with increased discharge. Age class abbreviations: ALL = all turtles, SI = small immature, LI = large immatures, A = adult-sized, ALL IMM = all immature sized animals (small + large), Large = all large turtles (large immatures + adult-sized). The time frame is reported in monthly ranges where responses were noted. The values reported in qAIC are the months with the most significant qAIC value.

Latitude	Age class	Cum-Whole		Cum-Bay		Non Cum-Whole		Non Cum-Bay	
		Time Frame	QAIC	Time Frame	QAIC	Time Frame	QAIC	Time Frame	QAIC
**-27**	ALL	8–12↑	12,11	9–12↑	12,11	1↑,7–9↑	8	1−,7–10↑	8
	SI	5–12↑	12,11	6–12↑	12,11	6–12↑	7,11,8,9	1↑,5–11↑	8,11
	LI	-	12,11,10	-	12,11,10	-	12,11,10	-	12,11,10
	A	8–12↑	12,11,10	8–12↑	12,11,10	7–9↑	8	7–10↑	8,9
	ALL IMM	7–12↑	12,11	8–12↑	12,11,10	6–11↑	7,8,11	1↑,7–11↑	11,18,12,10
	LARGE	9–11↑	12,11	9–12↑	12,11,10	7–9↑	8,12	7–10↑	12,10
**-25**	ALL	2↓,7–12↑	10,11	2–3↓,8–12↑	11,10,12	5–10↑	8	6–10↑	8
	SI	5–12↑	11,10,12	7–12↑	11,12,10,9	4–10↑	7,8	5–11↑	8,7,10
	LI	0–4↓,8–11↑	12,11,10	1–4↓	11,12,10	0–2↓,5–8↑	8,7,12	1↓,3↓,7–9↑	11,12,8
	A	0–3↓,8–12↑	12,10,11	0–2↓,8–12↑	11,10,12	0–1↓,6–9↑	8	0↓,6–10↑	7,8,9
	ALL IMM	6–12↑	10,11	7–12↑	11,12,10	4–10↑	7,8	5–10↑	8
	LARGE	0–4↓,8–12↑	12,11,10	0–4↓,8–12↑	11,12,10	0–2↓,5–9↑	8	0–1↓,6–9↑	8,7
**-23**	ALL	4–12↑	11,12	4–12↑	11,10,12	3–11↑	7	3–11↑	7,6
	SI	4–12↑	11,10	3–12↑	10,11,9	3–11↑	7	3–11↑	6,7
	LI	-	-	-	-	-	-	-	-
	A	0–12↑	12	7–12↑	11,12,10	0–1↑,6–12↑	10,9	7–11↑	7,8,10
	ALL IMM	4–12↑	11,10	4–12↑	11,10,12	3–11↑	7	3–11↑	6,7
	LARGE	0–12↑	12	7–12↑	11,12,10	0↑,5–12↑	10,8,9	6–12↑	8,7,10,11
**-19**	ALL	5–12↑	10,11	4–12↑	10,9	3–9↑	7,8	3–8↑	5
	SI	5–12↑	10,9,11	4–12↑	10,9,11,8	3–9↑	6,7,5	3–8↑	5,6
	LI	6–12↑	10,11,9	4–12↑	9,10,8,11	3–4↑,6↑,8–9↑	8,9	3–5↑	4,3,8,6
	A	-	11,12,10	4–12↑	10,11,9,8	3↑	3,	3–5↑	3,5
	ALL IMM	5–12↑	10,9	4–12↑	9,10,11,8	3–9↑	6,7,8	3–8↑	5,6
	LARGE	7–12↑	11,10,12	4–12↑	10,9,11,8	3↑	3	3–5↑	3,5

The exceptions for this patterns were the -25° block which showed a split response. The small immatures and all immature turtles within the -25° block did not show a split response, instead showed increased strandings with increasing discharge; the -19° and -23° blocks showed very similar response times to each other. The -25° and -27° blocks showed similar responses to each other (**Tables 10 and 11)**.

QAIC’s for all groups assessed were different and no patterns were observed (**Tables 10 and 11**). In most cases, the QAICs corresponded with significant responses, with an exception for the age classes which did not produce a significant relationship.

#### Peak discharge

**Table 12** summarizes the relationship between peak discharge and marine turtle stranding rates. In brief, it shows that in most cases, as peak discharge increased, the stranding rate also increased; each examined latitudinal block, there were similar stranding response times noted for embayments and the whole blocks for peak discharge; within each examined latitudinal block, similar response times for cumulative effect vs non-cumulative effect of peak discharge were observed.

**Table 12:** Model results for all turtles peak discharge. ↑ denotes increased strandings rates with increased discharge. ↓ denotes decreased stranding rates with increased discharge. Age class abbreviations: ALL = all turtles, SI = small immature, LI = large immatures, A = adult-sized, ALL IMM = all immature sized animals (small + large), Large = all large turtles (large immatures + adult-sized). The time frame is reported in monthly ranges where responses were noted. The values reported in qAIC are the months with the most significant qAIC value.

Latitude	Age class	Cum-Whole		Cum-Bay		Non Cum-Whole		Non Cum-Bay	
		Time Frame	QAIC	Time Frame	QAIC	Time Frame	QAIC	Time Frame	QAIC
**-27**	ALL	8–12↑	12,11	8–12↑	12,11	7–10↑	8,7	1↑,8–10↑	8,10,12
	SI	5–12↑	12,11	8–12↑	12,11	6–12↑	11,9	1↑,6↑,8–11↑	11,8,10
	LI	3–4↓,6↓	12,11,10	-	12,11,10	-	12,11,10	-	12,11,10
	A	7–12↑	12,11,10	8–12↑	12,11,10	7–9↑	8	7–10↑	8,9
	ALL IMM	7–12↑	12,11	8–12↑	12,11,10	7–11↑	1,7,12	8–11↑	11,10,12
	LARGE	9–12↑	12,11	9–12↑	12,11,10	7–9↑	8,12,11	7–10↑	12,10
**-25**	ALL	1–2↓,7–12↑	10,11	3↓,8–12↑	11,10,12	6–9↑	8,7	6–10↑	8
	SI	5–12↑	10,11	7–12↑	11,10,12,9	6–10↑	7,5	6–10↑	8,7,10
	LI	0–5↓,8–12↑	11,12,10	1–4↓,9–11↑	11,12,10	0–2↓,5–9↑	8	1↓,3↓,7–8↑	12,8,11
	A	0–5↓,8–12↑	12,11,10	0↓,9–12↑	10,11,12,9	0–1↓,7–9↑	8	7–10↑	8,7,9
	ALL IMM	7–12↑	10,11	8–12↑	11,12,10	5–10↑	7,8	6–10↑	8
	LARGE	0–5↓,8–12↑	12,11,10	0–5↓,9–12↑	11,12,10	0–2↓,6–9↑	8	1↓,7–9↑	8,7,12
**-23**	ALL	4–12↑	11,12	5–12↑	11,10,12	3–11↑	7	3–11↑	7,6
	SI	5–12↑	11,10	4–12↑	10,11,9,12	3–11↑	7	3–11↑	6,7
	LI	-	-	-	-	-	-	-	-
	A	0–12↑	12	8–12↑	11,12,10	0–1↑,6–12↑	10,9	7–11↑	7,10,8
	ALL IMM	5–12↑	11,10	4–12↑	11,10,12	3–11↑	7	3–11↑	6,7
	LARGE	0–12↑	12	8–12↑	11,12,10	0↑,5–12↑	8,10,7,9	6–11↑	10,7,8,11
**-19**	ALL	5–12↑	10,11	4–12↑	10,9	3–9↑	7	3–8↑	5
	SI	5–12↑	10,9	5–12↑	9,10,11,8	3–8↑	7,5,6	3–8↑	5
	LI	5–12↑	10,11,9,12	4–12↑	9,10,8	3–4↑,6↑,8–9↑	8,9	3–6↑	3,6,5,4
	A	5↑,7–12↑	11,10,12	3–12↑	10,9,11,8	3↑	3	3–5↑	3
	ALL IMM	5–12↑	10,9	5–12↑	9,10	3–9↑	6,7	3–8↑	5
	LARGE	5–12↑	11,10,9	3–12↑	9,10	3↑	3	3–5↑	3

The exceptions to these patterns were that the -25° block which showed a split response; small immature and all immature within the -25° block did not show a split response, instead showed increased strandings with increased discharge; large immatures within the whole -27° block showed a split response for cumulative effects and did not return significant responses for the non-cumulative effects or cumulative effects within the embayment; The -23° and -19° blocks showed very similar response times to each other (**Table 12**).

QAIC’s for all groups assessed were different and no patterns were observed (**Table 12**). In most cases, the QAICs corresponded with significant responses, with an exception for the age classes which did not produce a significant relationship.

#### Monthly mean maximum air temperature

**Table 13** summarizes the relationship between monthly mean maximum air temperature and marine turtle stranding rates. In brief, it shows most cases split responses were observed; when a split response was not noted stranding rates decreased and monthly mean maximum air temperature increased; there were similar response times noted for embayments and the whole blocks for monthly mean maximum air temperature; there were also similar response times for cumulative effect vs non-cumulative effect of monthly mean maximum air temperature.

**Table 13:** Model results for all turtles and monthly mean maximum temperature. ↑ denotes increased strandings rates with increased mean maximum temperature. ↓ denotes decreased stranding rates with increased mean maximum temperature. Age class abbreviations: ALL = all turtles, SI = small immature, LI = large immatures, A = adult-sized, ALL IMM = all immature sized animals (small + large), Large = all large turtles (large immatures + adult-sized). The time frame is reported in monthly ranges where responses were noted. The values reported in qAIC are the months with the most significant qAIC value.

Latitude	Age class	Cum-Whole		Cum-Bay		Non Cum-Whole		Non Cum-Bay	
		Time Frame	QAIC	Time Frame	QAIC	Time Frame	QAIC	Time Frame	QAIC
**-27**	ALL	1–9↓	6	1–9↓	6	0–5↓,7–11↑	9	1–4↓,7–11↑	8,9
	SI	0–9↓,11–12↓	5,4,6	0–12↓	6,7,5,4	0–4↓,6–10↑,12↓	2,8,9	0–4↓,7–10↑,12↓	8,9
	LI	1–9↓	6	1–8↓	12,11	1–5↓,7–11↑	9,8	1–4↓,6–10↑	10,12,9
	A	2–10↓	6,7	1–10↓	6,7	1–5↓,7–11↑	9,3	1–4↓,6–10↑	9,8
	ALL IMM	0–9↓,11↓	5,6,4	0–12↓	6,7	0–4↓,6–10↑,12↓	8,3,9	0–4↓,6–10↑,12↓	8,9
	LARGE	1–10↓	6	1–9↓	6,7,11	1–5↓,7–11↑	3,9	1–4↓,6–10↑	9,8,10
**-25**	ALL	1–8↓,11↑	5,4	2–8↓,10–12↑	11,5,12,6	1–4↓,6–10↑,12↓	8	1–4↓,7–11↑	9,8
	SI	0–5↓,10↑	3,2,10	3–7↓,11↑	11,12,5,6	0–3↓,5–9↑,11–12↓	7	1–4↓,7–10↑	9,8,10
	LI	2–9↓	5,6,4	2–8↓,11–12↑	11,12	1–5↓,7–11↑	3,9	1–4↓,7–11↑	9,11,10
	A	2–9↓	6,5	0↑,3–8↓,11–12↑	11,12,5,6	1–5↓,7–11↑	9	2–5↓,7–11↑	9,8,3
	ALL IMM	0–7↓	4,3,5	2–8↓,11–12↑	11,12,5,6	0–4↓,6–10↑,12↓	7,8	1–4↓,7–11↑	9,8
	LARGE	2–9↓	6,5	2–8↓,11–12↑	11,12,5,6	1–5↓,7–11↑	3,9	1–5↓,7–11↑	9,3
**-23**	ALL	1–12↓	5,4	0–12↓	12,4,3,5	1–4↓,7–9↑	2,3	1–3↓,6–8↑,12↓	2,12,1,3
	SI	0–8↓,11–12↓	3,2	0–12↓	12,4,3,5	0–3↓,6–9↑,12↓	2	0–3↓,6–8↑,12↓	2,1,12
	LI	-	-	-	-	-	-	-	-
	A	0↑,3↓,5–10↓	8,9	-	12,11,10	0↑,3–7↓,9–12↑	5	2–3↓	12,11,10
	ALL IMM	0–8↓,11–12↓	3,2	0–12↓	12,4,3	0–3↓,6–9↑,12↓	2	0–3↓,6–8↑,12↓	2,1,12,3
	LARGE	3–11↓	8,7,9	3–6↓		2–6↓,9–11↑	5,4	2–3↓	12,2,3,11
**-19**	ALL	0–12↓	12	0–12↓	12	0–3↓,7–8↑,11–12↓	12,1	0–3↓,6–7↑,11–12↓	12
	SI	0–12↓	12	0–12↓	12	0–3↓,6–8↑,11–12↓	1	0–3↓,6–7↑,11–12↓	1,12
	LI	0–12↓	12,11	0–6↓,9–12↓	12,11	0–3↓,12↓	12	0–2↓,12↓	12,1,2
	A	0–4↓,11–12↓	12	0–5↓,10–12↓	12	0–1↓,12↓	12	0–1↓,6↑,11–12↓	12
	ALL IMM	0–12↓	12	0–12↓	12	0–3↓,7–8↑,11–12↓	1	0–3↓,6–7↑,11–12↓	1,12
	LARGE	0–6↓,10–12↓	12	0–6↓,10–12↓	12	0–1↓,12↓	12	0–2↓,6↑,11–12↓	12

QAIC’s for all groups assessed were different and no patterns were observed (**Table 13**). In most cases, the QAICs corresponded with significant responses, with an exception for the age classes which did not produce a significant relationship.

#### Monthly mean minimum air temperature

**Table 14** summarizes the relationship between monthly mean maximum air temperature and marine turtle stranding rates. In brief it shows for cumulative effects across all latitudes, as monthly mean minimum air temperature increased, stranding rates for all turtles decreased; non-cumulative effects across all latitudes split responses were noted; non-cumulative effects across all latitudes, there was an immediate decrease in strandings rates (0-5-month lag), followed by an increase (5-10-month lag) and then a decreased (11-12-month lag); within each examined latitudinal block, there were similar response times noted for embayments and the whole blocks for monthly mean minimum air temperature; were very obvious stranding differences between cumulative and non-cumulative effects. Non-cumulative effects resulted in split responses whereas in most cases the cumulative effects resulted in decreasing stranding rate with increasing mean minimum air temperature.

**Table 14:** Model results for all turtles and monthly mean minimum temperature. ↑ denotes increased strandings rates with increased monthly mean minimum temperature. ↓ denotes decreased stranding rates with increased monthly mean minimum temperature. Age class abbreviations: ALL = all turtles, SI = small immature, LI = large immatures, A = adult-sized, ALL IMM = all immature sized animals (small + large), Large = all large turtles (large immatures + adult-sized). The time frame is reported in monthly ranges where responses were noted. The values reported in qAIC are the months with the most significant qAIC value.

Latitude	Age class	Cum-Whole		Cum-Bay		Non Cum-Whole		Non Cum-Bay	
		Time Frame	QAIC	Time Frame	QAIC	Time Frame	QAIC	Time Frame	QAIC
**-27**	ALL	1–9↓	6	1–9↓	6	1–4↓,7–11↑	9	1–4↓,7–11↑	8,9
	SI	0–8↓	4,5,6	0–9↓	6,5,7,4	0–4↓,6–10↑,11↓	8,9	0–4↓,6–10↑	8,9
	LI	1–9↓	6,7	1–9↓	12,11,10	1–4↓,7–11↑	9,9	0–4↓,7–10↑	10,12,9
	A	1–9↓	6	1–8↓	6,7	1–5↓,7–11↑	9	0–4↓,6–10↑	9,8
	ALL IMM	0–9↓	5,4,6	0–9↓	6,7	1–4↓,6–10↑,12↓	8,9	0–4↓,6–10↑	8,9
	LARGE	1–9↓,11↓	6	0–9↓	6,7	1–5↓,7–11↑	9	0–4↓,6–10↑	9,8,10
**-25**	ALL	0–8↓,12↓	4,5	1–10↓	5,6,4	0–4↓,6–10↑,12↓	2,8	1–4↓,7–10↑	9,2,8
	SI	0–6↓,12↓	12,2,3	1–10↓,12↓	5,6,4,7	0–3↓,5–8↑,11–12↓	12,7	1–4↓,7–10↑	9,2,8
	LI	1–8↓	5,4,6	0–9↓	12,7,11	1–4↓,6–10↑	8,9	1–4,7–10↑	9,10,8
	A	1–9↓	6,5,3	0–10↓	5,6,7,4	1–5↓,7–11↑	9	1–5↓,7–11↑	9,8,2
	ALL IMM	0–7↓,12↓	3,4,2	0–10↓,12↓	5,4,6,7	0–3↓,6–9↑,12↓	7,2,8	1–4↓,7–10↑	9,2,8
	LARGE	1–9↓	5,6	0–10↓	5,6,4,7	1–5↓,7–11↑	9	1–5↓,7–11↑	9,8
**-23**	ALL	0–12↓	5,4	0–8↓,10–12↓	12,3,2,4	0–4↓,7–10↑,12↓	2	0–3↓,6–8↑,12↓	2,1,7
	SI	0–7↓,11–12↓	3,2	0–4↓,11–12↓	2,3,12	0–3↓,5–9↑,12↓	1	0–3↓,6–8↑,12↓	1,2,7
	LI	-	-	-	-	-	-	-	-
	A	0↑,3↓,5–10↓	8,9	3–6↓,10–12↓	12,11,10	0↑,2–6↓,9–12↑	10,5,11	2–3↓	12,11,10
	ALL IMM	0–8↓,11–12↓	3,2	0–7↓,10–12↓	12,3,2,4	0–3↓,6–9↑,12↓	1,2	0–3↓,6–8↑,12↓	2,1,7
	LARGE	3–11↓	8,7,9	1–12↓	12,11,10	2–6↓,8–11↑	10	1–3↓,8↑	2,12,3,8
**-19**	ALL	0–6↓,12↓	12	0–5↓,12↓	12	0–2↓,6–8↑,12↓	12	0–2↓,5–8↑,11–12↓	12,0
	SI	0–6↓,12↓	3,2	0–5↓,12↓	2,1,3	0–3↓,5–9↑,12↓	7,1	0–2↓,5–8↑,11–12↓	12,1,0
	LI	0–6↓,12↓	12	0–3↓,12↓	12,11,2,3	0–2↓,8↑	12,8,9	0–2↓,6–7↑,12↓	12,6,7
	A	0–2↓,11–12↓	2	0–3↓,12↓	12	0–1↓,11–12↓	12	0–1↑,5–7↑,11–12↓	12,0,11
	ALL IMM	0–6↓,12↓	12,3,4,2	0–5↓,12↓	2,12,1,3	0–3↓,6–9↑,12↓	1	0–2↓,5–8↑,11–12↓	0,12,1
	LARGE	0–3↓,11–12↓	12	0–3↓,12↓	12	0–1↓,12↓	12	0–1↓,5–7↑,11–12↓	12

QAIC’s for all groups assessed were different and no patterns were observed (**Table 14**). In most cases, the QAICs corresponded with significant responses, with an exception for the age classes which did not produce a significant relationship.

#### Monthly average daily diurnal air temperature difference

**Table 15** summarizes the relationship between monthly average daily diurnal air temperature difference and marine turtle stranding rates. In brief it shows that in most cases, a split response was observed for monthly average daily diurnal air temperature difference; adults and large turtles from the whole -19° block did not show significant stranding responses; within each examined latitudinal block, there were similar response times noted for embayments and the whole blocks.

**Table 15:** Model results for all turtles and monthly average daily diurnal temperature difference. ↑ denotes increased strandings rates with increased average daily diurnal temperature difference. ↓ denotes decreased stranding rates with increased average daily diurnal temperature difference. Age class abbreviations: ALL = all turtles, SI = small immature, LI = large immatures, A = adult-sized, ALL IMM = all immature sized animals (small + large), Large = all large turtles (large immatures + adult-sized). The time frame is reported in monthly ranges where responses were noted. The values reported in qAIC are the months with the most significant qAIC value.

Latitude	Age class	Cum-Whole		Cum-Bay		Non Cum-Whole		Non Cum-Bay	
		Time Frame	QAIC	Time Frame	QAIC	Time Frame	QAIC	Time Frame	QAIC
**-27**	ALL	1–8↑	6	1–8↑	6	1–4↑,7–11↓	9,8	1–4↑,7–11↓	8,9
	SI	0–6↑	12,11	1–6↑,10–11↓	6,7,5,4	0–3↑,6–10↓	8,9	1–4↑,6–11↓	8,9
	LI	1–8↑	12,6,11,7	0–8↑	12,11	1–4↑,7–11↓	9	0–3↑,8–9↓	9,12,11
	A	1–7↑,11–12↑	6,12,5	0–7↑,11−	6,7	1–4↑,7–11↓	9,8	1–3↑,6–10↓	8,9
	ALL IMM	0–7↑	4,5,3	0–7↑	6,7	0–4↑,6–11↓	8,9	0–4↑,6–11↓	9,8
	LARGE	1–8↑	6,4,5	0–8↑	6,7,11	1–4↑,7–11↓	9	0–3↑,6–10↓	9
**-25**	ALL	0–8↑,12	2,3	0–12↑	5,4,3,2	0–3↑,6–9↓,12↑	2,1	0–4↑,7–10↓,12↑	2
	SI	0–6↑,12↑	2,1,0	0–12↑	4,5,3	0–2↑,5–8↓,11–12↑	12	0–4↓,8–9↓,12↑	12,0,1
	LI	0–7↑	2,3,4	1–9↑,12↑	12,11,4	0–3↑,6–10↓,12↑	8,7,9	0–4↓,6–10↓,12↑	12,9,8
	A	0–8↑	4,5,3	0–12↑	5,4,6,3	0–4↑,7–10↓,12↑	9,8,2	0–5,7–9↓,12↑	2
	ALL IMM	0–7↑	2	0–12↑	4,3,5,2	0–3↑,5–9↓,12↑	12,7,1	0–4↑,7–10↓,12↑	12,2,9,1
	LARGE	0–8↑	3,4,2	0–12↑	5,4,3,2	0–4↑,6–10↓,12↑	8,9	0–4↑,7–10↓,12↑	2
**-23**	ALL	0–4↑	12,11,10	0–2↑	2,12	0–1↑,5–9↓	8,9,5,6	0–1↑,5↓	12,9,5,7
	SI	0–3↑	12,11,10	0–2↑,9–12↓	12,11,10	0↑,5↓	5	0↑,5↓,7↓,9↓12↓	5,9,12
	LI	-	-	-	-	-	-	-	-
	A	11–12↓	12	2–6↑	11,12,10	3–4↑,6–11↓	10,9,8,12	2↑	2,12,11
	ALL IMM	0–3↑	2,12,1	0–2↑,10–12↓	12,11,10	0–1↑,5–6↓	5	0↑ 5–7↓,9↓,12↓	9,7,12
	LARGE	2–4↑	12	1–5↑	12,11,3	1↑,6–10↓	8,10,9,12	2↑	2,10,12,11,9
**-19**	ALL	0–3↑,8–12↓	10,11	0–2↑,7–12↓	10,9	0–2↑,5–9↓	8,7	0–1↑,4–8↓	7,6
	SI	0–3↑,7–12↓	10,11	0–3↑,7–12↓	10,11,9	0–2↑,5–9↓	7	0–1↑,4–9↓	7,6
	LI	10–11↓	11,12,10	8–11↓	10,9,11,8	6↓,8↓	8,9,6	5–6↓	6,8,5,7
	A	-	12,9,11,10	0↑,7–10↓	8,9,10,11	-	12,11	0↑,5–6↓	5,11,7
	ALL IMM	0–3↑,7–12↓	10,11	0–3↑,7–12↓	10,9,11	0–3↓,5–9↓	8,7	0–1↑,4–9↓,12↑	6,7
	LARGE	-	10,11,9	0↑,7–11↓	9,10,8	-	12,8	0↑,4–7↓	5,6,7

QAIC’s for all groups assessed were different and no patterns were observed (**Table 15**). In most cases, the QAICs corresponded with significant responses, with an exception for the age classes which did not produce a significant relationship.

## Discussion

This is the first study of its kind to elucidate the effects of individual environmental variables on the stranding rates of coastal marine turtle populations and provides a baseline for future predictive models that can be used as real-time management tools. We found that strandings occurred after a lag phase, with water discharge having the greatest effect on stranding numbers. This study found that the cumulative effects of freshwater discharge in all latitudes resulted in increased strandings 7–12 months later (**Tables 3–5, Tables 10–12)**. The cumulative effects of mean maximum and minimum air temperature resulted in decreased stranding rates immediately through to a lag of 9 months (**Tables 6 and 7, Tables 13 and 14**). Monthly average daily diurnal air temperature difference resulted in increased strandings immediately through to a lag of 8 months (**Tables 8–15**). There was an overall decrease in stranding rates 2–8 months after high rainfall events, although the relationship was less clear (**Tables 2–9**).

When comparing cumulative effects against non-cumulative effects, non-cumulative effects were more likely to produce split or dual responses **(Tables 2–15).** This could be due to cumulative effects having a more lasting, stronger effect. The cumulative effect of multiple months of increased discharge and rainfall potentially does not allow time for the seagrasses to recover and hence have a stronger effect on marine turtles through their diets.

When analyzing latitude along the Queensland coastline, there was no evidence that stranding rates were different in different latitude, although there were some noticeable differences **(Tables 2–15).** When comparing the effect of latitude for discharge, the -25° block produced split responses whereas the other blocks produced single responses (**Tables 3–5, Tables 10–12)**. Although stranding rates at different latitudes responded differently the overall pattern of lagged stranding was similar, suggesting the increase in marine turtle stranding was not just a local issue rather, at least, a state-wide issue that occurred and warranted consideration at a state or larger regional level. Given the migratory pattern of marine turtles and their ability to move within the broader range of their individual home sites [39], mitigation needs to consider widespread impacts and not just local habitats of known marine turtle populations.

When analyzing age classes across the variables, there were no observed patterns in relation to which group responded first for each variable **(Tables 2–15)**. This was not expected, it was expected that small immatures would be more susceptible to changes in dietary availability and would show responses before other age classes.

Embayments, when compared to the whole latitudinal block, did not influence the pattern of strandings but did decrease the lag phase for each examined environmental variable **(Tables 2–15).** This could indicate that embayments are areas of concentrated discharge which is not dissipating in to the wider area, thus having an increased negative effect on the turtles and the aquatic vegetation for which they depend.

An interesting outcome from this study was that, while the response trends were the similar, green turtles as a group tended to strand ~ 1 month earlier than the all turtles group **(Tables 2–15).** The reasons for this earlier response were uncertain, but the authors postulated that this may be related to diet. However, the small sample sizes of the other species prevented this trend being statistically analysed further.

As with any exploratory modeling, we identified several limitations that may influence the accuracy of any developed model including distributed sample equality, equal adequate sample sizes for each species, availability of environmental data such as seagrass abundance, habitat type and the distance offshore that an event was recorded. One of the limitations of these models is that the stranding sample size was different for each examined latitudinal block. Larger sample size may make the relationships more noticeable than smaller sample sizes, but as this used one of the longest running and largest datasets available, this may be difficult to correct. The -27° block recorded the most number of strandings over the study period (**Table 1**). This latitudinal block encompasses Moreton Bay which is known to support large fields of seagrass and other aquatic vegetation. The -23° block recorded the least number of strandings over the study period for a recognized hotspot (**Table 1**).

The model may have been strengthened by the use of food availability/viability as a factor. However, due to the paucity of data, it was decided to use weather as a proxy to this as weather data is available in immediate time. There is evidence that discharge and rainfall are adequate proxies for seagrass abundance as large-scale seagrass die-off have been closely associated in time and intensity to flooding [40–42]. This study may also have been strengthened by determining if different species showed different responses times and directions. This was not possible due to the small sample sizes of the other species occurring within the study location.

Within coastal waters green turtles are almost exclusively herbivorous, feeding principally on seagrass and a wide range of algae and mangrove fruits [43,44]. Occasionally, green turtles feed on macroplankton, including jellyfish, bluebottles, small crustaceans and dead fish [43,44]. Brand-Gardner et al. [45] found that within Moreton Bay small immature green turtles forage selectively on plants with higher nitrogen levels and lower levels of fiber (such as *Gracilaria* sp.). Due to this strong dependency on aquatic vegetation, it has meant that green turtles living within inshore coast habitats where aquatic vegetation is a large component of their diet have suffered during and post the extreme weather events, such as the flooding in Queensland in 2010–11.

This study has identified that there are relationships between specific environmental variables (freshwater discharge and air temperature) and marine turtle strandings. These findings will allow first responders to be more prepared for increases in strandings following increases in freshwater discharge rates. These models can be used to form the basis for an exploratory model which can be used to predict future responses to adverse weather events including increased freshwater discharge, increased rainfall and changes in mean air temperature.
